# Collaborative and secure transmission of medical data applied to mobile healthcare

**DOI:** 10.1186/s12938-019-0674-x

**Published:** 2019-05-20

**Authors:** Weimin Chen, Zhigang Chen, Fang Cui

**Affiliations:** 1 School of Computer Science and Engineering, Central South University, Changsha, 410075 China; 2 School of Information and Electronic Engineering, Hunan City University, Yiyang, 413000 China

**Keywords:** Mobile healthcare, Opportunistic networks, Medical data, Circle of friends, Integer wavelet transform

## Abstract

**Purpose:**

We propose a collaborative and secure transmission scheme in order to safely and efficiently transmit medical data and provide telemedicine services, lighten the load on wireless access networks, and improve the quality of medical treatment such as surgery.

**Methods:**

First, the transmission technology based on opportunistic networks is used to upload patient physiological data and share medical information. Second, we propose a trusted transfer scheme based on the circle of friends, which is constructed with historical encounters and social features of nodes. This scheme takes the forwarding policy of each packet by close friends to effectively prevent the participation of strangers, and avoid privacy issues and deal with selfish behaviors. At the same time, the structure of friend circle is beneficial to the improvement of medical data transmission. Third, we present a lossless compression scheme with less computation and higher compression ratio to reduce the amount of medical data and improve the performance of the transmission.

**Results:**

The experimental results show that the proposed scheme is effective and has good transmission performance while ensuring the safety and reliability of media data.

**Conclusion:**

The mobile healthcare faces some challenges such as the vastness of medical data and sensitivity of patient information. Using opportunistic networks to transmit medical data in mobile healthcare is a good solution, which can effectively divert and offload the data traffic of mobile Internet. The structure of friend circles and the technology of data compression are beneficial to safely and efficiently transmit the patient’s physiological parameters and medical health information.

## Introduction

**Fig. 1:**
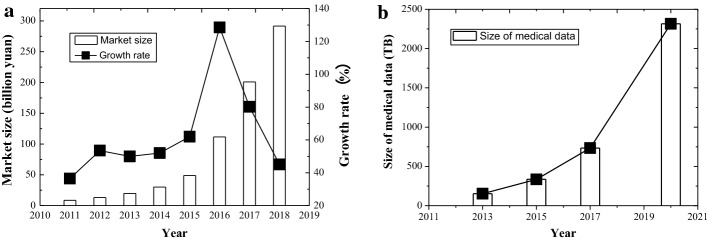
Development of mobile healthcare. **a** market size in China, **b** global medical data size

With the development of society and the improvement of living standard, people have a higher demand for medical services and health management. Mobile healthcare (mHealth), also known as mobile health, can provide medical services and information via the use of mobile communication technologies, such as tablets and smartphones. It gives an effective solution way for people to listen to the advice of doctors or access to a variety of health-related information (including physical examination, health care, disease assessment, medical treatment, and rehabilitation) whether at home or on the road. Alleviating the difficulty of getting medical treatment, reducing medical costs, improving the level of diagnosis and treatment, and promoting health and disease prevention, mHealth has become a hot topic in academia and industry recently [1]. In China, from the perspective of market size, there was about 2.95 billion yuan in 2014 and increased by 44.7% to 4.27 billion yuan in 2015, as shown in Fig. 1a. From the aspect of user size, they reached 72 million in 2014 and 138 million in 2015. In addition, there are more than 2000 mHealth platforms.

However, mHealth faces the following problems in the application. First, it is regarding how to process the massive medical data. The diagnosis and treatment of patients will generate a lot of information, including their personal information, past medical history, examination items, hospitalization records, and discharge records. According to the statistics of the second Xiangya hospital of the Central South University, each patient undergoing PET-CT examination will produce about 300 images, a total of about 1 GB of data. Unfortunately, massive images and videos generated by mobile intelligent terminals have overwhelmed the current mobile Internet, and their rapid growth rate has far exceeded the speed of the expansion of mobile Internet bandwidth [2]. The yearbook report of Cisco also shows that video data account for more than 85% of the data traffic of the entire mobile Internet in 2018 [3]. Second, it expresses concerns about the patient privacy and data security. Mobile healthcare is highly dependent on network and information technology, and it is difficult to ensure the security of patients’ personal information and medical information. In the process of data transmission, data anomalies and leakage problems will be caused by the external malicious interference. In the interview, 25% interviewees expressed concerns about patient privacy and data security. It can be seen that the privacy and security of data are the focus and difficulty for both patients and medical workers.

Therefore, mHealth needs a secure and efficient data transmission technology. The opportunistic networks (OppNets) do not need a complete connecting path between nodes. It uses the encounter opportunity formed by node movement to realize communication in the scheme of “store-carrying-forward,” which has many advantages such as flexible networking, rapid expansion, and distributed control [4]. In particular, with the development of communication technology in recent years, mobile intelligent terminals have been rapidly popularized. Using these devices to network, OppNets can realize conveniently, quickly, and efficiently the sharing of content, resources, and services. The emergence of OppNets promotes the process of free communication in medical data sharing environment, expands the use range of network, and is an important part of ubiquitous communication in the future [5].

In this paper, the OppNets is used to transmit medical data in mHealth applications. To improve the performance of transmission, and avoid the privacy issue and the selfish behavior of nodes, we propose a novel trust transmission scheme based on friend circles in OppNets for mHealth. This scheme utilizes the historical contacts and social character of nodes to construct the friend circles in order to create a collaborative and secure transmission environment, and selects a node as the relay only if it satisfies the following conditions: it is within the friend circles of the source node, and has more opportunity to access the destination node. By forwarding each packet via friends, this mechanism can prevent the strangers from participating in the transmission, and avoid significantly privacy issues and the selfish behavior. At the same time, it has high transmission performance because of the structure of friend circles.

The contributions of this paper mainly include the following items. (1) To lessen the cost pressure on users and also lighten the load on wireless access networks, we introduce OppNets into mHealth systems. (2) To achieve higher transmission performance and deal with the selfishness and privacy issues, we propose a collaborative and secure medical data transmission scheme based on friend circles. (3) To reduce the amount of medical data in the transmission, we propose a lossless compression scheme with less computation and higher compression ratio.

## Related work

Mobile healthcare based on medical data communication technology and intelligent terminal has become a new telemedicine mode, and it has moved from a concept to a reality which its application extends to every field of medical treatment [6]. Doukas et al. [7] present a mHealth system by means of Cloud Computing. In [8], a mHealth service system is introduced by means of RFID technology and mobile devices. David et al. [9] present mHealth applications and discuss possible challenges facing the development of mobile applications. Baig et al. [10] analyze the critical issues and challenges related to security and privacy of data in mobile phone-based sensor applications of mHealth. Rongxing et al. [11] introduce a secure and privacy-preserving framework based on a new access control and privacy-preserving technique. Kumar et al. [12] propose a novel solution of security of private data transmission. Rahman et al. [13] discuss the security scheme to prevent the attack of wireless communications in mHealth systems. Azzedine et al. [14] propose a secure multicast strategy to only permit trustworthy nodes to take part in communications. AlMuhtadi et al. [15] propose an emergency call mechanism with a view to preserving personal privacy. Kuan et al. [16] present many secure and privacy-preserving strategies in mHealth.

OppNets is ubiquitous because of the use of mobile smart terminals, and has the characteristics of node mobile and self-organization, which helps to have a good application prospect in various normal and nonnormal scenarios and attract the attention of the domestic and foreign academic circles. First, many transmission algorithms are proposed to improve the performance of OppNets. Vahdat et al. [17] propose a flooding-based Epidemic algorithm with the highest transmission success rate and lowest latency. Lindgren et al. [18] use the historical encounters to present a predictive transmission algorithm PRoPHET. Hui et al. [19] propose the BUBBLE algorithm which uses the community structure to forward the data packet. Wenrui et al. [20] propose the ferrying mechanism to deliver the message by the ferry node. Second, some trust transmission methods are proposed to deal with the selfishness and privacy issues. In addition, many routing algorithms based on other characteristics of nodes are proposed, such as the relationship [21], interest [22], context aware [23], Big Data [24–27], IoMT [28, 29], etc. Na et al. [30] selected the appropriate node to forward messages by counting the number of feedbacks to measure the trust values of node based on the “watchdog” mechanism. In [31], the selfish nodes were bypassed according to the trust values of node which can be evaluated through the number of historical encounters. In [32], the trust metric of each node was the number of hops from it to the destination on forwarding path, and the data were forwarded sequentially from the lower trust nodes to the higher trust nodes. Trifunovic et al. [33] proposed an OppNets trust model according to social trust which was evaluated by the relationship between nodes, and it was established by the network topology and the number of hop. Xi et al. [34] proposed a forwarding algorithm according to social trust which is built by the similarity, the intimacy and the service between nodes. Bulut et al. [35] introduced the metric of trust, and presented a routing algorithm which used friendships to make the forwarding decisions of messages.

In the existing mHealth research, wireless access network and mobile terminal are mainly used to transmit medical data and provide telemedicine services. At the same time, privacy protection and secure transmission are discussed in terms of security technology and means. However, the current mobile Internet has been overburdened, and the data traffic needs to be diverted and offloaded. Therefore, exploring the data transmission technology of OppNets and applying it to mobile medical service is of great significance to promote the application of mHealth.

## Methodology

### System model

In 1967, a social psychologist Milgram Stanley put forward the Six Degrees of Separation theory [36]. It reveals an important principle: any two strangers in the world can contact with each other by a link made up of six people. Later, he conducted a “chain letters” experiment in which he randomly sent some letters to 160 people living in Omaha, and asked each recipient to send the letter to a friend who thought he was closer to a Boston stockbroker. In the end, most of the letters were sent to the stockbroker after five or six steps. Motivated by the Six Degrees of Separation phenomenon, we propose a trusted data-forwarding scheme based on friend circles. This scheme can effectively prevent strangers from participating in the process of data transmission by constructing a trusted transmission environment-friend circles and relying on the friends of nodes to forward each packet. Therefore, the privacy problem and selfish behavior can be avoided effectively, and the data transmission performance is high.

**Fig. 2:**
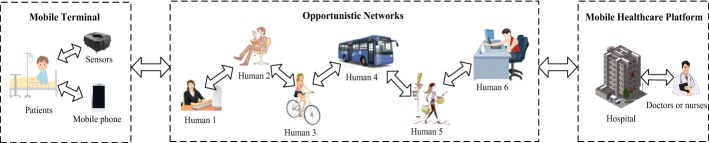
Model of our mobile healthcare system

In our work, the model of mHealth system is designed as shown in Fig. 2. Based on the transmission of OppNets, the patient physiological data collected by various sensors are sent to the hospital in order to realize the personal monitoring of human health, and patients can access whenever and wherever a variety of medical health information from doctors or other peoples to establish a health concept oriented to promoting disease prevention and strengthening disease prevention.

In the OppNets, we assume that each node has different social relations and behaviors which can be described by his/her social features and history encounters. These social features can be obtained by a certain means (for example, questionnaire survey) before the deployment of the network, and the history encounters can also be collected with the wireless terminal devices after a period of network running.

The transmission mechanism in mHealth is described as follows: (1) Transmission of the patient physiological data. To ensure the security, these data use a single-path transmission scheme. That is, they are forwarded in turn by the people who are within the friend circles of the previous one and have more opportunity to access the destination. (2) Transmission of the health information accessed from other people. To improve the transmission efficiency, this information uses a multipath transmission scheme. In other words, they are copied into all friend circles of the people, and the process ends when the people obtain this information.

### Transmission algorithm based on friend circles

#### Construction of friend circles

In the section, we first analyze the relationship between nodes reflected by their historical encounters, and discuss the importance of different social features of nodes, then construct the friend circles of nodes according to this information.

##### Historical encounters

The historical encounters can generally be obtained from the records of software attached to the mobile intelligent terminal. They can reflect the relationship between nodes and reveal the characteristics of node mobility over a period of time by some statistical measurements, including the number, average time, and average time intervals of encounters, and so on. In general, the more the number of encounters between nodes is, the longer their encounter time is, and the higher their encounter frequency is, the closer their relationship is. On the contrary, the less their encounter number is, the shorter their encounter time is, and the lower their encounter frequency is, the more distant their relationship is.To exactly measure the relationship between nodes, we introduce a metric as follows:

1 $$ w_{ij} = \frac{T}{{\int_{0}^{T} {f(t)dt} }} = \frac{2T}{{t_{1}^{2} + t_{2}^{2} + \cdots + t_{n}^{2} }}{\kern 1pt} {\kern 1pt} {\kern 1pt} = \frac{2T}{{\sum\limits_{k = 1}^{n} {t_{k}^{2} } }}, $$

where *T* is the time interval between the collected data in system. *f*(*t*) is the average wait time for each packet forwarding. *t*_*k*_ is the *k*th interval time of encounters. *w*_*ij*_ is the measurement of the relationship between nodes *v*_*i*_ and *v*_*j*_. It is obvious that *w*_*ij*_≥ 1, and the value of *w*_*ij*_ is bigger, nodes *v*_*i*_ and *v*_*j*_ are closer.

##### Social features

In OppNets, nodes are the smart mobile devices used or carried by people, so they have the social characteristics of people, including natural features (such as gender, age, and body mass index) and social features (such as classmate, friend, and colleague). These features can be used to describe the relationship between nodes in society, and affect node movement and data forwarding. It is proved that the smaller the feature distance between nodes is, the more their connection is [37]. However, there are many social features of nodes. Two real trace datasets (Infocom 2006 [38] and MIT Reality [39]) provide more than 10 social features, such as affiliation, city, neighborhood, research group. In these features, only a small fraction has a significant impact on the relationship between nodes, and can be picked out by their Shannon entropy as follows:

2 $$ E(f_{i} ) = - \sum\limits_{j = 1}^{{k_{j} }} {p(x_{j} )\log (x_{j} )}, $$

where *x*_*j*_ is a possible value of the social feature *f*_*i*_. *p*(*x*_*j*_) is the probability of *x*_*j*_. Clearly, the larger the Shannon entropy *E*(*f*_*i*_) is, the bigger the impact of *f*_*i*_ is.

##### Circles of friend

###### **Definition 1**

For any two nodes in the network, if their relationship metric is greater than a certain threshold, namely, they have a close relationship, then they can be called friend. It is described as follows:

3 $$ F_{i} = \left\{ {v_{j} |w_{ij} > \tau } \right\} \quad j = 1,2, \ldots ,n, $$

where *w*_*ij*_ is the relationship metric defined in formula (2). *τ* is the threshold which is used to adjust the degree of intimacy relationship between nodes. *F*_*i*_ is the friend set of node *v*_*i*_.

###### **Definition 2**

For any nodes in the network, if they have the same value of a social feature (that is, they have the same hobbies and characteristics) and they are friends with each other, they form a circle of friends.

The specific construction process of friend circles is as follows:

Step 1. Construct the social circle of the node according to its social features.

4 $$ C_{i} = \left\{ {v_{j} |f_{i} \in F(v_{j} )} \right\}{\kern 1pt} {\kern 1pt} {\kern 1pt} {\kern 1pt} {\kern 1pt} {\kern 1pt} {\kern 1pt} {\kern 1pt} {\kern 1pt} {\kern 1pt} {\kern 1pt} {\kern 1pt} {\kern 1pt} {\kern 1pt} {\kern 1pt} {\kern 1pt} {\kern 1pt} j = 1,2, \ldots ,n, $$

where *f*_*i*_ is the *i*th feature value of the node. *F*(*v*_*j*_) is a function for finding the feature values of node *v*_*j*_. *C*_*i*_ is the *i*th social circle of the node and is composed of nodes with *f*_*i*_. It is worth mentioning that there are many social features for each node, and each feature has many values. In practical application, important social features and eigenvalues should be screened according to the formula (2) to avoid interfering with the process of data transmission by the irrelevant social features.

**Fig. 3:**
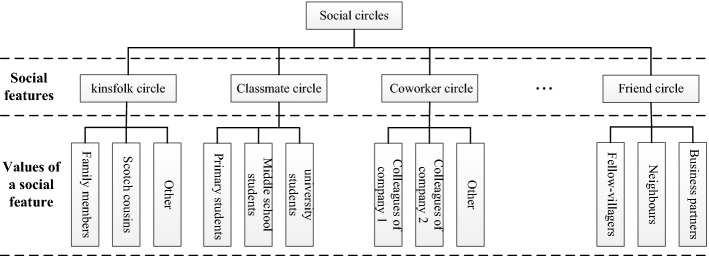
Schematic diagrams of the social circles

The social circles of nodes are shown in Fig. 3. Here, a social feature corresponds to a certain kind of social circle, and a value of the social feature corresponds to a specific social circle.

Step 2. The friend circles are constructed by removing strangers from the social circles of the node using the formulas (3) and (4). Thus, we have

5 $$ FC_{i} = C_{i} \cap F_{i}, $$

where *FC*_*i*_ is a friend circle of node *v*_*i*_.

#### Trust transmission algorithm

In the section, to meet the needs of different application scenarios of mHealth, we provide two trust transmission algorithms based on the structure of friend circles.

##### Multipath transmission algorithm

In OppNets, a flooding-based routing algorithm, such as epidemic [17], is proposed. It has the highest delivery ratio and minimum transmission delay by sending a large number of data copies along multiple paths. Based on the above idea, we propose a multipath transmission algorithm based on the structure of friend circles. Its transmission strategy is designed as follows: the source node forwards a copy of data to each of its friend circles, and each recipient does the same until the destination node receive the data. Forwarding via the friends of the node, the data are transmitted along the multiple circles of friends, which can improve the chance of meeting the destination node as much as possible, and obtain a larger delivery ratio and a smaller transmission delay. This algorithm is suitable for the application scenarios where the data need to be transmitted quickly and widely. For example, the medical and health information that patients need is transmitted in mHealth.

The multipath transmission algorithm based on friend circles is shown in algorithm 1, its process is explained as follows: At a certain point, the node *v*_*c*_ has a data packet *p* to send to the node *v*_*d*_, and it meets the node *v*_*i*_. If *v*_*i*_ is *v*_*d*_, or *v*_*i*_ does not have *p* and is a member in friend circles of *v*_*c*_, *v*_*c*_ copies *p* to *v*_*i*_. If *v*_*d*_ has accepted *p*, this transmission process ends. Otherwise, the above process is repeated.
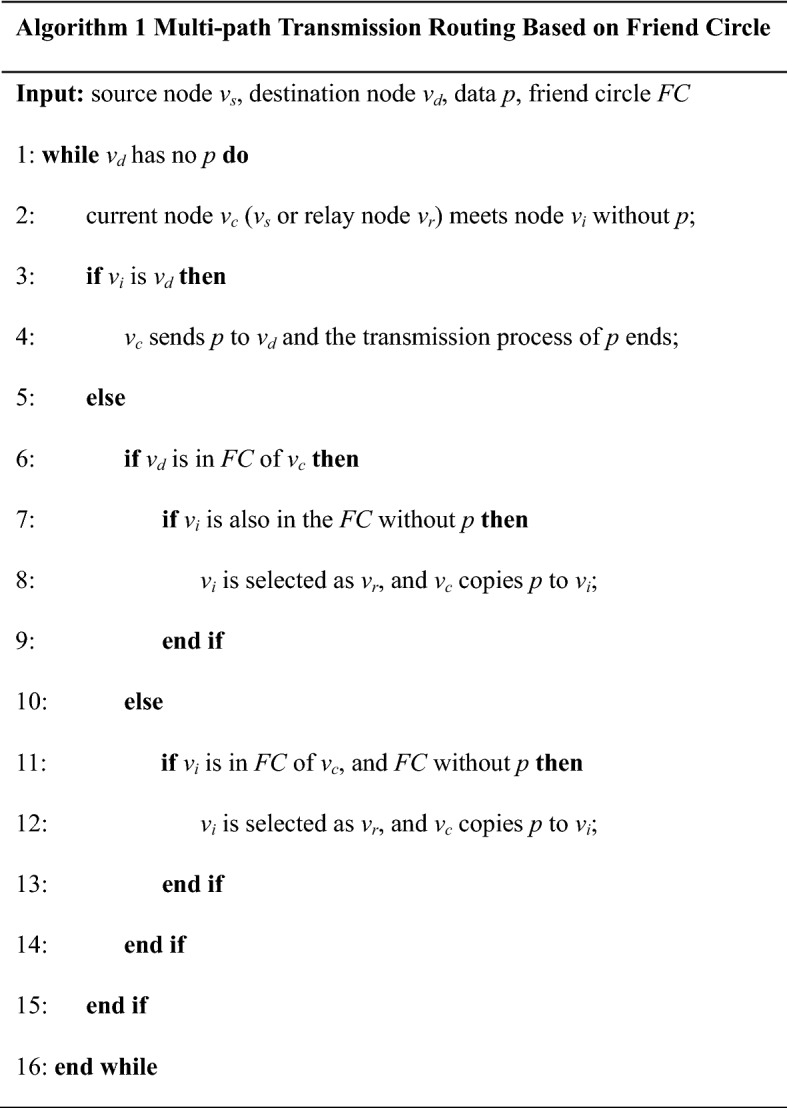


##### Single-path transmission algorithm

In order to reduce the network overhead and ensure the security of data, we propose a single-path transmission algorithm based on friend circles. In this algorithm, only one piece of data is allowed to exist in the whole transmission process, and the data are transferred along a path and finally arrive at the destination node.

In order to improve the forwarding effect, the relay nodes need to be selectively identified. Therefore, we designed a transmission algorithm based on the greedy strategy. If the encounter node is a member in the friend circles of the current node, and it has more opportunities to access the destination node, it can be selected as the relay node. Furthermore, if the encounter node is in the friend circles of the destination node, it is considered to have more opportunities to access the destination node. In addition, if the encounter node has more friends than the current node, it is considered to have more opportunities to access the destination node. In a word, the selection strategy of relay node is as follows: the encounter node is a member in the friend circles of the current node. If it is in the friend circles of the destination node, or it has more friends than the current node, it is selected as the relay node.

The single-path transmission algorithm based on friend circles is shown in algorithm 2, its process is explained as follows: At a certain point, the node *v*_*c*_ has a data packet *p* to be sent to the node *v*_*d*_, and encounters the node *v*_*i*_. If *v*_*i*_ is $v_{d}$, or *v*_*i*_ has more opportunities to access *v*_*d*_ than *v*_*c*_, *v*_*i*_ is select a relay and *v*_*c*_ forwards *p* to *v*_*i*_. If *v*_*d*_ has accepted *p*, this transmission process ends. Otherwise, the above process is repeated.

The algorithm can only forward the data packet to an optimal encountered node which is within the friend circles of the current node and considered to have more opportunities to access the destination node. It is similar to the experiment of chain letter, that is, it can verify the Six-Degree Separation theory to a certain extent. Therefore, this algorithm has the minimum number of data copies and good transmission performance. It is suitable for the application scenario with dense distribution of nodes and high-security requirements. For example, the patients’ physiological data are uploaded to the hospital in mHealth.
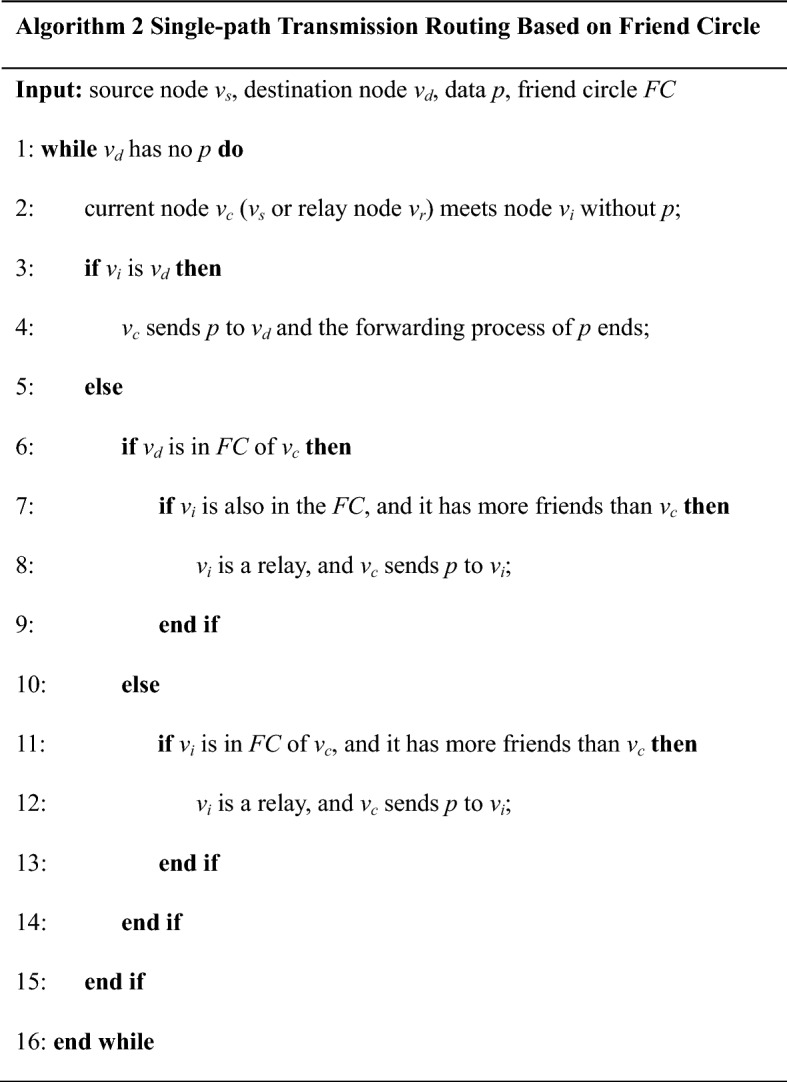


### Data lossless compression

#### Description of problem

In the diagnosis and treatment of patients, we produce a large number of medical data, such as patient information, medical record, examination data, doctor’s advice, etc. Among them, the examination data are especially large. To get an accurate understanding of the patient’s condition, various examinations are often required. In the laboratory inspection, it includes routine, biochemical, immunologic, bacteriological, and other tests; each examination contains a number of subitem checks; and each check contains medical data of a hundred fields. Table 1 shows a routine biochemical test report which contains 26 inspection items, and more items are examined in some special cases. In the imaging examination, it includes X-ray, CT, B-ultrasound, NMR, and these examinations will produce a lot of images. In the case of PET-CT, each patient produces an average of 400 images, of about 2 GB.

**Table 1:** Biochemical examination report

NO.	Inspection item	Unit	Reference ranges
1	ALT	U/L	0.0–41.0
2	AST	U/L	0.0–40.0
3	AST/ALT		0.8–2.0
4	AIP	U/L	45–135
5	r-GT	U/L	0.0–47.0
6	TBIL	Umol/L	6.0–20.5
7	DBIL	Umol/L	0.0–6.0
8	IBIL	Umol/L	1.7–13.8
9	TP	g/L	60.0–80.0
10	ALB	g/L	35.0–55.0
11	GLB	g/L	20.0–30.0
12	TG	mmol/L	0.70–1.70
13	CHO	mmol/L	2.30–5.70
14	HDL	mmol/L	0.77–2.25
15	LDL	mmol/L	2.30–3.30
16	GLU	mmol/L	3.90–6.40
17	BUN	mmol/L	1.7–8.3
18	UA	Umol/L	140–428
19	Cr	Umol/L	53–115
20	CO2CP	mmol/L	22–31
21	HBsAg		–
22	HBsAb		–
23	HBeAg		–
24	HBcAb		–

According to the report [40], the global medical big data were 153 TB in 2013, and is expected to reach 2314 TB by 2020, estimated at an annual growth rate of 48%, as shown in Fig. 1b. Faced with such a large amount of data, the communication technology is not enough to deal with them. Therefore, a compression technique is needed to reduce the amount of medical data in the network.

#### Integer wavelet transform

In image processing, the input data are expressed as integers, so we use the integer wavelet transform to compress medical image data. Integer wavelet transform can remove the correlation between data to a certain extent, eliminate redundant information, and reduce the entropy of data; thus, it can provide a theoretical basis for lossless data compression [41, 42].

For the original signal *S*_*i*_, it is decomposed into the low-frequency signal *S*_*i*−1_ and the high-frequency detail signal *D*_*i*−1_ by integer wavelet transform, and the transformation process contains the following three steps.

Step 1. Splitting: The original signal *S*_*i*_ is usually divided into two disjoint subsets: even and odd sequences.

6 $$ splite\left( {S_{i} } \right) = \left( {even_{i - 1} ,odd_{i - 1} } \right) = \left( {S_{i - 1} ,D_{i - 1} } \right) $$

Step 2. Prediction: In view of the correlation between data, *S*_*i*-1_ can be used to predict *D*_*i*−1_ by adopting a prediction operator *p*, so that *D*_*i*−1_ = *p*(*S*_*i*−1_). One of the simplest prediction operators *p* is the mean of two adjacent even numbers, we have:

7 $$ D_{{i - 1,{\kern 1pt} j}} = S_{i,2j + 1} - \left\lfloor {{{\left( {S_{i,2j} - S_{i,2j - 2} } \right)} \mathord{\left/ {\vphantom {{\left( {S_{i,2j} - S_{i,2j - 2} } \right)} 2}} \right. \kern-0pt} 2}} \right\rfloor $$

Step 3. Updating:

8 $$ S_{{^{{_{i - 1,j} }} }} = S_{i,2j} + \left\lfloor {\left( {D_{i - 1,j} + D_{i - 1,j - 1} } \right)} \right\rfloor $$

**Fig. 4:**
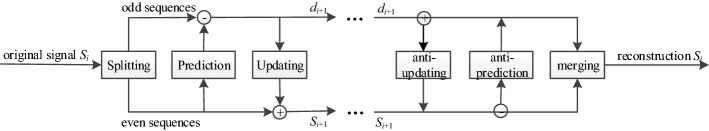
Process of integer wavelet transform

After *n* times decomposition, the wavelet of the original data is expressed as {*S*_*i*−*n*_, *D*_*i*−*n*_, *D*_*i*−*n*+1_,…, *D*_*i*−1_}. The process of reconstruction can recover the original signal by anti-updating, antiprediction, and merging steps, as shown in Fig. 4.

#### Entropy code

To improve the compression performance, the data need to be rescheduled and shuffled before entropy coding to prioritize the same bytes as possible. In the compression process, the algorithm of entropy code is the deflate algorithm which is a general lossless compression algorithm. It is a combination of the lz77 dictionary coding and the Huffman coding [43]. In addition, TS wavelet filter is chosen to carry out integer wavelet transform, and its advantage is that the compression and decompression time overhead is much less and the compression ratio is slightly less than the binary arithmetic coding.

### Simulation configuration

#### Simulation datasets

Two real trace datasets are used in our simulations, and their details are described as follows. (1) Infocom 2006 trace datasets [38] is a common trace data and available at Crawdad. This datasets is collected by recording the contacts between attendees with iMote. It consisted of two kinds of data: contacts data and social features of the attendees. Among them, the data of 61 attendees are only used because the profiles of other 18 attendees have some problems. We use the data including 74,981 contacts in 337,418 s, and five social features such as affiliation, living city, nationality, language, and residing country. (2) MIT reality mining datasets [39] is a common trace data and collected by recording the contacts between 94 teachers and students with mobile phone. It is also consisted of two kinds of data: contacts data and social features. Among them, we use the data of 57 participants because the incomplete information of the other, including 411,313 contacts in 897,921 s, and five social features such as neighborhood, commuter time, haunting, affiliation and working time.

#### Performance metrics

There are four performance metrics used to assess each algorithm. (1) Delivery ratio: the ratio of the number of medical data delivered successfully to send out during a given interval. (2) Delivery delay: the time taken for the data to be successfully delivered. (3) Hop count: the number of nodes through which the data was successfully sent. (4) Number of forwarding: the number of data being forwarded during transmission.

#### Simulation method

Several transmission algorithms are used for comparison in the simulations. (1) Multipath transmission algorithm based on friend circles (TAFC-M): each node copies the message to all encounter nodes if they are the members of the friend circles of the current node. (2) Single-path transmission algorithm based on friend circles (TAFC-S): a node is forwarded only if it is within the friend circles of the current node and has more opportunity to access the destination. (3) Spray-and-wait (S-W) [44]: there are 10 data copies at the beginning. First, each node with more than one copy sends half to the encounter node, and then none of the nodes forwards any data copy until the destination is encountered. (4) SimBet [45]: it forwards data based on SimBet utility. (5) ST-Epidemic (ST-E) [32]: an effective transmission algorithm based on social trust. (6) F-R [33]: an effective transmission algorithm based on friendship.

## Results

### Data compression

In the simulation, the proposed algorithm is evaluated by comparing to several lossless compression schemes. Among them, TS wavelet filter is selected to carry out integer wavelet transform, and the actual data MIT-BIH (Beth Israel hospital of Massachusetts institute of technology) arrhythmia database [46] is used as simulation data. The results are shown in Table 2, it can be seen that the proposed compression algorithm increases the compression ratio by 55% compared with several compression algorithms, while the compression time cost is equal to that of other compression algorithms. The simulation shows that the proposed compression algorithm based on integer wavelet transform has proven excellent performance.

**Table 2:** Comparison of several compression algorithms

Algorithms	Compression time (s)	Decompression time (s)	File size (Byte)	Compression ratio
Deflate	0.51	0.21	1,669,346	3.08
Bzip2	10.55	1.91	1,634,557	3.15
Ucl	2.04	0.18	1,519,459	3.56
Our algorithm	0.63	0.26	1,629,369	4.97

### Effect of threshold

**Fig. 5:**
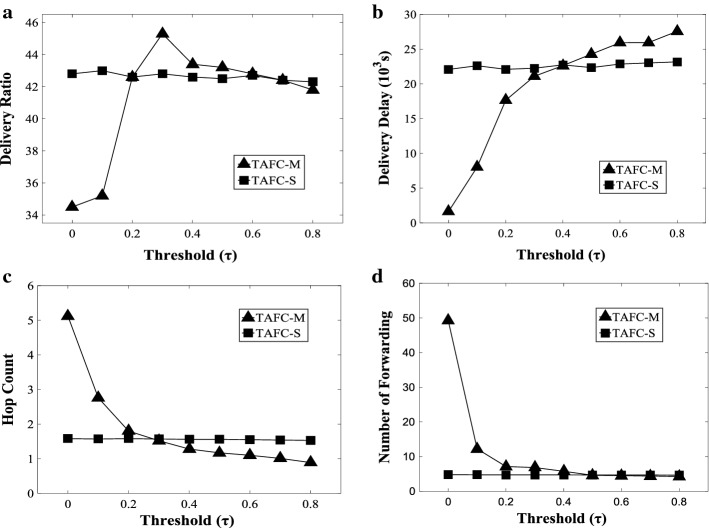
Effect of threshold *τ* **a** delivery ratio, **b** delivery delay, **c** hop count, **d** number of forwarding

In the simulation, the effect of threshold *τ* is evaluated. Figure 5 shows that *τ* has important influences on the multipath scheme, while the effect of the single-path scheme is relatively small. In the multipath scheme, with the threshold *τ* increases, the lists of nodes friend get smaller, and nodes which participate in the forwarding get smaller. Hence, the number of forwarding gets smaller, the hop count gets smaller, and the delivery delay rises. To our surprise, the delivery ratio increases maximum and then decreases. The reason is as follows: the threshold decreases to a certain value, the copies of the message is so enough that the cache is full and some packages are discarded. Hence, the delivery ratio reduces.

### Comparison of different transmission algorithms

**Fig. 6:**
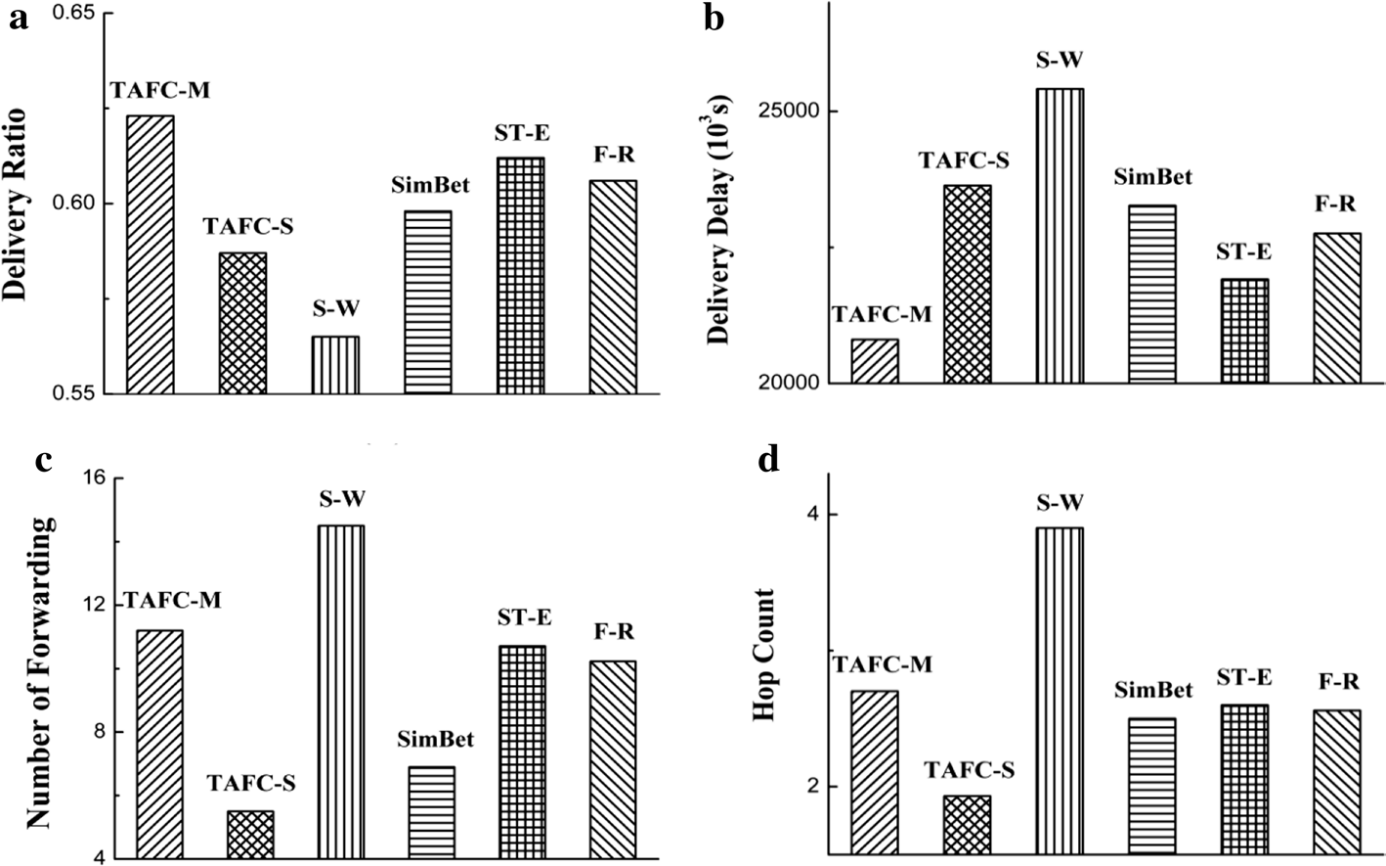
Comparison of several transmission algorithms in Infocom 2006 trace datasets, **a** delivery ratio, **b** delivery delay, **c** number of forwarding, **d** hop count

In the simulations, the proposed transmission algorithms are assessed by comparing to server existing algorithms. From Fig. 6, it is clear that the multipath scheme has a larger delivery ratio and a shorter delay. It can achieve 62% of delivery ratio, while single-path, SimBet, S-W, ST-E, and F-R, could only deliver 58%, 59%, 56%, 61.2%, and 60.6% respectively. In addition, the single-path scheme has the least hop counts and number of forwarding. Compared to multipath scheme, SimBet, S-W, ST-E, and F-R, the single-path scheme decreases the number of forwarding by about 50.9%, 20.3%, 62.1%, 48.6%, and 46.3% respectively.

In the MIT reality mining datasets, the simulations result is shown in Table 3. Clearly, our algorithm is better than other. Compared to the single-path, SimBet, S-W, ST-E, and F-R, the multipath scheme increases the delivery rate by about 7.3%, 5.1%, 10.2%, 2.8% and 4.0%, and reduces the latency by about 5.5%, 3.6%, 7.2%, 2.0% and 2.4% respectively. Compared to the multipath, SimBet, S-W, ST-E, and F-R, the single-path scheme decreases number of forwarding by about 55.8%, 29.7%, 65.4%, 54.4%, and 54.3% respectively. The simulation results are consistent with the above results.

**Table 3:** Comparison of several transmission algorithms in MIT reality mining datasets

Performance metric	TAFC-M	TAFC-S	S-W	SimBet	ST-E	F-R
Delivery ratio	0.807	0.752	0.732	0.768	0.785	0.776
Delivery delay (s)	82,249	87,043	88,639	85,345	83,924	84,238
Forwarding number	10.84	4.79	13.84	6.82	10.52	10.47
Hop count	3.2	1.9	3.9	2.5	3.1	3.0

## Discussion

In previous studies, wireless access networks such as 3G/4G and Wi-Fi were mainly used to transmit medical data in mHealth. However, with the vigorous development of the mobile Internet, its data traffic is growing exponentially, which brings serious challenges to divert and unload these traffic and has become a common concern of academia and industry. The introducing of OppNets can not only lighten the load of access networks, but also reduce the cost pressure of users. Therefore, it is a significant attempt to introduce OppNets into mHealth.

In our study, the transmission algorithm based on friend circles has a low time complexity and only *O*(*n*). Its disadvantage lies in the use of the social features and historical encounters of nodes. The former can be obtained by filling in a questionnaire when the user is registered, and the latter is a kind of dynamic data which need to be updated and maintained every now and then. In addition, the data compression algorithm based on integer wavelet transform is a mature and developed technology, which has the advantages of consuming less computation and less compression times.

In the transmission algorithm, the parameter *τ* is mainly used to control the degree of intimacy between nodes. The larger the value of *τ* is, the closer the relationship between friends is, and the fewer the number of nodes involved in medical data transmission is, the higher the data security is, but the greater the transmission delay of medical data is. On the contrary, the smaller the value of *τ* is, the more the number of nodes participated in transmission is, the lower the medical data security is, and the shorter the transmission delay of medical data is.

In this study, we mainly use the friend circles to achieve the safe and efficient medical data transmission. Among them, the definition of friends only takes into account historical encounter data, not other factors, such as recommendations of others, and it can be carried out to more accurately measure credibility between nodes in the future. In addition, we only consider simply the data compression problem, and we should construct a complete data compression scheme in the future from the perspectives of time and space.

## Conclusion

The mHealth framework faces some challenges such as the vastness of medical data, sensitivity of patient information and ubiquity of patient physiological information collection, whereas OppNets has the characteristics of node mobile, self-organization, and ubiquitous, which makes it has a good application prospect in various normal and nonnormal scenarios. In this paper, we use OppNets to transmit medical data in mHealth, which is a good solution, and can effectively divert and offload the data traffic of mobile Internet. In addition, the structure of friend circles is beneficial to safely and efficiently transmit the patient’s physiological parameters and medical health information, and the data compression based on the integer wavelet transform can effectively reduce the amount and size of data and is beneficial to the faster transmission of medical data. The experimental results show that the proposed scheme is effective and has good transmission performance while ensuring the safety and reliability of media data.

## Abbreviations

mHealth: mobile healthcare
OppNets: opportunistic networks
CT: computed tomography
PET-CT: positron emission tomography/computed tomography
TAFC-M: multipath transmission algorithm based on friend circles
TAFC-S: single-path transmission algorithm based on friend circles
S-W: spray-and-wait
ST-E: ST-Epidemic
F-R: effective transmission algorithm based on friendship
MIT-BIH: Beth Israel hospital of Massachusetts institute of technology
